# Incidence of Peripheral Arterial Disease and Its Association with Pulse Pressure: A Prospective Cohort Study

**DOI:** 10.3389/fendo.2017.00333

**Published:** 2017-11-24

**Authors:** Yong Mao, Yixiang Huang, Haining Yu, Peng Xu, Guangping Yu, Jinming Yu, Yiqiang Zhan

**Affiliations:** ^1^Department of Epidemiology and Health Statistics, School of Public Health, Kunming Medical University, Kunming, China; ^2^School of Public Health, Sun Yat-sen University, Guangzhou, China; ^3^Shandong Cancer Hospital Affiliated to Shandong University, Shandong Academy of Medical Sciences, Jinan, China; ^4^Wuqing Center for Disease Control and Prevention, Tianjin, China; ^5^Department of Health Education and Health Behavior, School of Public Health, Fudan University, Shanghai, China; ^6^Karolinska Institutet, Stockholm, Sweden

**Keywords:** pulse pressure, peripheral arterial disease, ankle brachial index, blood pressure, cohort study

## Abstract

**Background:**

The association of pulse pressure and peripheral arterial disease (PAD) has seldom been examined using a prospective design. This study aimed to investigate the association of pulse pressure with PAD incidence in an elderly general population.

**Methods:**

We utilized data from a cohort conducted in Beijing with additionally 2-year follow-up time. PAD was defined as an ankle brachial index value <0.9 in either leg. Cox proportional hazard regression model was used to quantify the magnitude of pulse pressure on PAD incidence.

**Results:**

During a 2-year follow-up time, 357 of 4,201 (8.5%) participants developed PAD with 105 (6.9%) men and 252 (9.4%) women, respectively. After adjusting for baseline age, sex, body mass index, hypertension, diabetes, total cholesterol, and high-density lipoprotein cholesterol, and smoking, the hazard ratio and 95% confidence interval for people with pulse pressure greater than 60 mmHg was 2.20 (1.53, 3.15) compared with those whose pulse pressure was less than 40 mmHg. A linear trend was observed for the association of pulse pressure with PAD.

**Conclusion:**

Higher pulse pressure was associated with higher PAD incidence.

## Introduction

Peripheral arterial disease (PAD) is a common circulatory problem in which narrowed arteries reduce blood flow to limbs (1, 2). It is also a strong predictor for cardiovascular diseases, mortality, and stroke, independent of traditional cardiovascular risk factors (3, 4). Identification of those with PAD has profound public health and clinical implications for earlier disease prevention and treatment (5).

A number of risk factors have been identified for PAD risk assessment, such as smoking, diabetes, height, and dyslipidemia (6–9). Previous investigations suggested higher pulse pressure may be an independent predictor for PAD and this relationship was observed in several studies (10–12). However, most of these studies were based on cross-sectional design or case–control setting in which the temporal order was difficult to establish due to the weakness of study design. A prospective investigation of the association between pulse pressure and PAD remains scarce.

The present study aimed to examine the incidence of PAD, and to assess its association with pulse pressure in a general senior population.

## Patients and Methods

### Study Design and Participants

This study was a prospective cohort with participants originally recruited for chronic diseases and risk factors assessment in Beijing in 2007 with additional 2-year follow-up until 2009. A more detailed study design was described previously (13, 14). The present study included participants who were 50 years and over. In brief, 5,885 participants from 38 communities were recruited. During the 2-year follow-up duration, 1,095 participants emigrated or relocated, 402 refused to attend the follow-up examination, and 19 died. Of the remaining participants, 164 had baseline PAD and 4 had missing data on core covariates; the final analysis included 4,201 people who were free of PAD at baseline. All participants provided written informed consent to attend this study and subsequent follow-up examination, and the study protocol and ethical approval was obtained from the Ethic Committee of Beijing Municipal Science and Technology Commission.

### Measurements

The health interview was performed by trained medical staff at community clinics using a well-established questionnaire to determine demographic and behavioral characteristics of the study population. Information regarding birthday, gender, and smoking status were collected. Physical examination included anthropometric measurements, blood pressure, and medical history. Height and weight were measured to the nearest 0.1 cm and 0.1 kg, respectively, with the subject standing barefoot in light clothes. Waist circumference was measured to the nearest 0.1 cm at the mid-point between the 12th rib and right anterior superior iliac spine. Body mass index (BMI) was calculated as weight (kilogram) to be divided by square of height (meter). Blood pressure was measured using standard mercury sphygmomanometer on the right arm in sitting position after the participants rested for 5 min. Phase 1 and phase 5 Korotkoff sound was used as systolic blood pressure (SBP) and diastolic blood pressure (DBP), respectively. Blood pressure was measured twice with the average results for the data analysis. Medical history was obtained from medical record and confirmed by local general practitioners.

Blood samples were collected from all the participants after an overnight fasting. All the biochemical measurements were conducted in the central laboratory of Peking University People’s Hospital. Concentrations of fasting glucose, total cholesterol (TC), high-density lipoprotein cholesterol were measured using an auto analyzer (Hitachi 717, Hitachi Instruments, Inc., Tokyo, Japan). Hypertension was defined as SBP ≥140 mmHg, DBP ≥90 mmHg, or current medication for hypertension, and diabetes mellitus was defined as fasting glucose ≥7.0 mmol/L or current medication for diabetes.

### Determination of Pulse Pressure and PAD

Pulse pressure was calculated as the SBP minus the DBP. The ankle brachial index (ABI) was determined complying with a standard protocol. After 5 min of rest, a standard mercury sphygmomanometer and a Doppler stethoscope with 5 mHz probe (Nicolet, Elite 100 R, 5 mHz probe, USA) were used to determine the bilateral brachial, tibial, and dorsal arteries with participants in supine position. Measurements were carried out twice and averaged for analysis. ABI was calculated as the ratio of the highest SBP in the leg to the highest SBP in the arm. The lower value of ABIs was used as the patient-specific ABI for analysis. ABI <0.9 was considered as PAD (15).

### Statistical Analysis

Continuous variables were presented as mean ± SD and categorical variables were presented as frequencies and proportions. In the descriptive analysis, we present the basic characteristics of study subjects in men and women separately. Then in the exploratory analysis, we examined the association between pulse pressure and PAD using Cox proportional hazard regression models. Before treating pulse pressure as a continuous variable, pulse pressure was categorized into quartiles with the lowest quartiles as the reference. Three models were used for the analysis. The first model only included pulse pressure followed by the second model adjusted for age and gender as confounders. The third model was further adjusted for BMI, high-density lipoprotein cholesterol, TC, hypertension, diabetes, and smoking status. Hazard ratios (HRs) with 95% confidence interval (CI) were also presented. Kaplan–Meier method was used to estimate the PAD-free probability for different pulse pressure groups. All the analyses were two-tailed and *P* < 0.05 was considered to be statistically significant. All the statistics were obtained using R 3.1.

## Results

### Basic Characteristics

Among the 4,201 study participants, 1,527 (36.3%) were men and 2,674 (63.7%) were women. As shown in Table 1, the mean age was 61.1 (SD, 7.7) for men and 60.4 (SD, 7.8) for women. The mean ABI was 1.09 (SD, 0.08) with 1.11 (SD, 0.09) for men and 1.07 (SD, 0.08) for women. Tables 2 and 3 described the characteristics by PAD status. During a median 2-year (range: 1.8–2.3 years) follow-up time, 357 (8.5%) participants developed PAD with 105 (6.9%) men and 252 (9.4%) women corresponding to 33.9 per 1,000 person-year in men and 45.5 per 1,000 person-year in women, respectively.

**Table 1:** Baseline characteristics of study participants.

Variables	Men (*n* = 1,527)	Women (*n* = 2,674)
Age (years)	61.1 ± 7.7	60.4 ± 7.8
SBP (mmHg)	132.7 ± 18.7	132.4 ± 19.6
DBP (mmHg)	81.7 ± 10.7	80.5 ± 10.1
ABI	1.11 ± 0.09	1.07 ± 0.08
BMI (kg/m^2^)	24.9 ± 3.5	26.0 ± 4.0
Glucose (mmol/L)	5.21 ± 1.75	5.29 ± 1.71
TC (mmol/L)	4.75 ± 0.92	5.21 ± 0.93
Hypertension, *n* (%)	657 (43.0)	1,092 (40.8)
Diabetes, *n* (%)	133 (8.7)	251 (9.4)
Smoking, *n* (%)	847 (55.5)	267 (10.0)
Medication (excluding anti-thrombotic), *n* (%)	216 (14.1)	405 (15.1)
Anti-thrombotic, *n* (%)	288 (18.9)	459 (17.2)

*SBP, systolic blood pressure; DBP, diastolic blood pressure; ABI, ankle brachial index; BMI, body mass index; TC, total cholesterol*.

**Table 2:** Characteristics of study participants at follow-up.

Variables	Men (*n* = 1,527)	Women (*n* = 2,674)
Age (years)	63.16 ± 7.73	62.46 ± 7.77
SBP (mmHg)	134.35 ± 20.05	133.54 ± 19.88
DBP (mmHg)	82.71 ± 11.44	80.17 ± 10.46
ABI	1.08 ± 0.12	1.04 ± 0.11
BMI (kg/m^2^)	27.68 ± 58.23	26.95 ± 16.01
Waist (cm)	89.44 ± 10.34	87.48 ± 10.34
Hip (cm)	96.83 ± 6.85	98.11 ± 8.32
Glucose (mmol/L)	5.47 ± 2.01	5.5 ± 1.89
TC (mmol/L)	4.51 ± 0.84	4.97 ± 0.85
Hypertension, *n* (%)	788 (51.6)	1,273 (47.6)
Diabetes, *n* (%)	163 (10.7)	275 (10.3)
Smoking, *n* (%)	774 (50.7)	240 (9.0)
Medication (excluding anti-thrombotic), *n* (%)	198 (13.0)	376 (14.1)
Anti-thrombotic, *n* (%)	192 (12.6)	360 (13.5)

*SBP, systolic blood pressure; DBP, diastolic blood pressure; ABI, ankle brachial index; BMI, body mass index; TC, total cholesterol*.

**Table 3:** Characteristics of study participants who developed peripheral arterial disease at follow-up.

Variables	Men (*n* = 105)	Women (*n* = 252)
Age (years)	66.45 ± 8.07	67.1 ± 9.35
SBP (mmHg)	138.39 ± 19.53	138.08 ± 21.65
DBP (mmHg)	82.19 ± 10.15	79.79 ± 10.54
ABI	0.81 ± 0.08	0.81 ± 0.1
BMI (kg/m^2^)	25.19 ± 4.15	26.33 ± 4.21
Waist (cm)	89.45 ± 11.31	88.57 ± 10.18
Hip (cm)	96.83 ± 7.76	98.35 ± 10.59
W/H ratio	0.92 ± 0.06	0.9 ± 0.08
Glucose (mmol/L)	5.38 ± 1.61	5.59 ± 2.01
TC (mmol/L)	4.71 ± 0.92	5.11 ± 1.07
Hypertension, *n* (%)	57 (54.3)	145 (57.5)
Diabetes, *n* (%)	12 (11.4)	23 (9.1)
Smoking, *n* (%)	54 (51.4)	29 (11.5)
Medication (excluding anti-thrombotic), *n* (%)	16 (15.2)	41 (16.2)
Anti-thrombotic, *n* (%)	15 (14.3)	38 (15.1)

*SBP, systolic blood pressure; DBP, diastolic blood pressure; ABI, ankle brachial index; BMI, body mass index; TC, total cholesterol*.

### Association between Pulse Pressure and PAD Incidence

**Figure 1:**
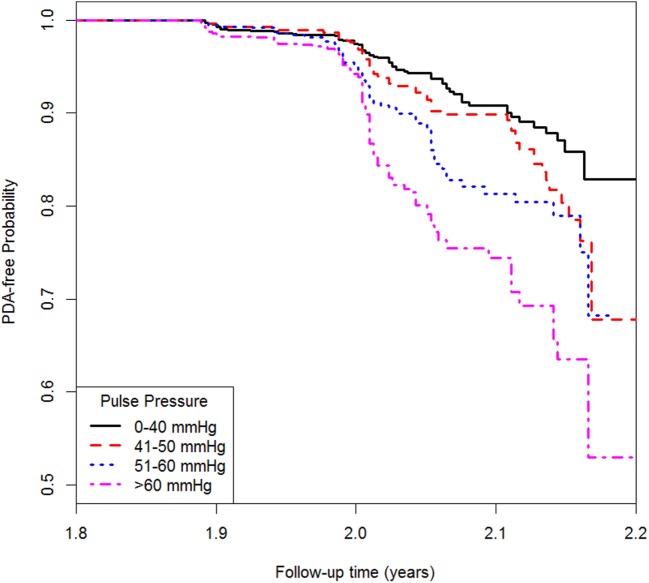
Kaplan–Meier plot for peripheral arterial disease by pulse pressure.

Table 4 shows the association between pulse pressure and PAD. Model 1 only included pulse pressure, while Model 2 was adjusted for age and gender followed by Model 3 adjusted for age, gender, BMI, hypertension, diabetes, TC, high-density lipoprotein cholesterol, and smoking. In Model 3, each 10 mmHg increase in pulse pressure was associated with 19% higher risk of PAD when treating pulse pressure as a continuous variable. The HR (95% CI) for people with pulse pressure greater than 60 mmHg was 2.20 (1.53, 3.15) compared with those whose pulse pressure was less than 40 mmHg. Similar result was shown from the Kaplan–Meier plot in Figure 1.

**Table 4:** Association between pulse pressure and peripheral arterial disease incidence, hazard ratio (95% confidence interval).

Pulse pressure	Model 1	Model 2	Model 3	Model 4
0–40 mmHg	1.0	1.0	1.0	1.0
41–50 mmHg	1.38 (0.99, 1.90)	1.22 (0.88, 1.69)	1.25 (0.90, 1.73)	1.25 (0.90, 1.73)
51–60 mmHg	1.99 (1.45, 2.74)	1.59 (1.15, 2.20)	1.63 (1.17, 2.27)	1.63 (1.17, 2.27)
>60 mmHg	3.25 (2.42, 4.38)	2.10 (1.53, 2.87)	2.20 (1.53, 3.15)	2.19 (1.53, 3.14)
Continuous	1.30 (1.23, 1.38)	1.17 (1.10, 1.25)	1.18 (1.10, 1.28)	1.18 (1.10, 1.28)

*Model 1 included only pulse pressure; Model 2 was further adjusted for age and sex; Model 3 additionally adjusted for body mass index, hypertension, diabetes, total cholesterol, high-density lipoprotein cholesterol, and smoking. Model 4 was additionally adjusted for medication use*.

## Discussion

In the present study, we examined the incidence of PAD and its association with pulse pressure in prospective cohort. We found that higher pulse pressure was associated with high risk of PAD and this association was independent of several risk factors including age, gender, high-density lipoprotein cholesterol, TC, hypertension, diabetes, and smoking status.

There are few studies of PAD incidence based on ABI. The Limburg study reported PAD incidence rate was 17.8 in men and 22.9 in women per 1,000 person-year at the age of 65 and over (16) which is lower than our study. The difference may be due to the different study population, follow-up time, or socioeconomic status. Several observational studies have been conducted to examine whether pulse pressure is associated with PAD (17, 18). One study from Finland reported one 1 mmHg increase of pulse pressure to be associated with 1.03 odds ratio of PAD which was approximately 1.34 odds ratio for 10 mmHg increase of pulse pressure (11). Our previous study also found 5-mmHg increase in pulse pressure was associated with a 17 and 15% increase of PAD risk in men and women (10). A more recent study from Japan observed an odds ratio for PAD was 1.30 for increasing pulse pressure (12). However, all of these studies are based on cross-sectional design or in a case–control setting. It might be difficult to determine if it is pulse pressure affect PAD or the opposite. Our current study, by using the prospectively collected data, found 10 mmHg to be associated with 19% higher risk for 2-year PAD incidence risk. This finding is consistent with published studies while has more strength in terms of its longitudinal cohort design and avoid reverse causation.

In this study, we observed women had higher incidence of PAD compared with men, which was also observed in The Limburg Study (15). Several factors might contribute to the sex difference in PAD incidence. Firstly, women were less physically active on than men nowadays in China, especially for the elderly (19). Physical activity was a protective factor for PAD; less physical activity could contribute to higher PAD incidence. Secondly, previous studies also revealed sex hormone and menopausal status were associated with lower risk of cardiovascular diseases for women. However, this phenomenon was not observed for PAD. In the Framingham Heart Study, sex hormone concentrations were not associated PAD risk in neither pre-menopausal nor post-menopausal women (20). Thirdly, heart rate was reversely correlated with ABI (21) and women tended to have higher heart rate and heart rate variability than men (22). However, more research is needed to clarify this sex inequality and more attention should be paid to women with higher risks of PAD.

Several potential molecular mechanisms might explain the observed association between pulse pressure and PAD. Pulse pressure was found to impair vascular endothelial function (23), and persistence of endothelial dysfunction could lead to arterial stiffness, atherosclerosis, and PAD (24, 25). Additionally, the *ENPP1* Q121 variant could increase pulse pressure and reduced insulin signaling (26). Insulin resistance was strongly and independently associated with PAD (27). Moreover, pulse pressure promotes the apoptosis of vascular smooth muscle cell and this effect is independent of endothelial and mitogen-activated protein kinase (28). The synergistic effects of all these processes and lipid accumulation (29) accelerate the narrowing of the arteries in the lower extremities and aggravate intermittent claudication.

In order to examine if the observed association was independent of traditional risk factors of PAD, we additionally adjusted for several variables in our analyses. In the multivariable adjusted models, the magnitude of pulse press on PAD incidence was attenuated compared with the unadjusted models. Two reasons might explain this effect size decrease. These adjusted factors, such as age and gender, might be the confounders for both PAD and pulse pressure; thus, the HR will decrease after removing the confounding effects by model adjustment. Other risk factors, such as hypertension, diabetes, or lipid fractions, might mediate the effect from pulse pressure to PAD. However, these explanations need to be further tested in future studies. A few studies examined the relationship between ABI and flow-mediated vasodilation and found they were strongly correlated (30). However, we did not measure flow-mediated vasodilation in our current study.

### Limitations

Although the data used in our study was collected prospectively, the median follow-up time was only 2 years. In this study, we only considered several important confounders in the analyses; a residual confounding could still bias the observed association. Additional analysis by collecting more variables, such as genetic variants (31) and other biomarkers (32–34), should be useful. The present study was conducted in an Asia population; the generalization of the results to other ethnic groups may be limited.

## Conclusion

Higher pulse pressure was associated with PAD incidence, and this association might be mediated by several mechanisms.

## Ethics Statement

This study was carried out in accordance with the recommendations of Ethic Committee of Beijing Municipal Science and Technology Commission with written informed consent from all subjects. All subjects gave written informed consent in accordance with the Declaration of Helsinki. The protocol was approved by the Ethic Committee of Beijing Municipal Science and Technology Commission’.

## Author Contributions

YM, YZ, HY, and JY designed this study. YZ and GY analyzed these data. All authors drafted this manuscript.

## Conflict of Interest Statement

The authors declare that the research was conducted in the absence of any commercial or financial relationships that could be construed as a potential conflict of interest.
